# Introduction and validation of the Natural Disasters Picture System (NDPS)

**DOI:** 10.1371/journal.pone.0201942

**Published:** 2018-08-08

**Authors:** Gaëtan Merlhiot, Martial Mermillod, Jean-Luc Le Pennec, Laurie Mondillon

**Affiliations:** 1 Université Clermont Auvergne, CNRS, Laboratoire de Psychologie Sociale et Cognitive, Physiological and Psychosocial Stress, LABEX ClerVolc, Clermont-Ferrand, France; 2 Université Grenoble Alpes, LPNC, Grenoble, France; 3 Institut Universitaire de France, Paris, France; 4 CNRS, UMR 5105 LPNC, Grenoble, France; 5 Université Clermont Auvergne, CNRS, IRD, OPGC, Laboratoire Magmas et Volcans, LABEX ClerVolc, Clermont-Ferrand, France; University of Zurich, SWITZERLAND

## Abstract

Given the growing demand for studies dealing with natural disasters, the research fields of emotion and social cognition require validated picture stimuli of natural hazards. Such material is essential for studying perceptual processes and behaviors of exposed individuals, and it could find practical applications, such as the improvement of communication strategies during crises. We present the Natural Disasters Picture System (NDPS), a database of pictures of natural hazards, with an emphasis on volcanic threats, and their impact on the environment and humans. We first describe in detail the picture selection and database creation. We then report the validation procedure. One hundred twenty participants rated the pictures on the basis of four dimensions: valence, arousal, dominance and certainty. For each picture, we ultimately determined the best-fitting emotion on the basis of its dimensional pattern. The Hierarchical Ascendant Classification, which yielded 4 clusters subdivided into 9 classes, indicated a highly consistent and distinctive classification of the pictures. Overall, 90% of the pictures elicited negative emotions (fear or sadness), and the other 10% induced neutral to positive emotions (e.g., aesthetic emotions). The NDPS offers a new tool for studying natural events and disasters in the field of affective and cognitive sciences, which will benefit from scientific research and its practical applications. The NDPS is unrestrictedly accessible for researchers.

## Introduction

There is a growing demand for studies dealing with natural disasters in the research field of emotion and social cognition. This inquiry, however, requires validated stimuli of natural hazards. Thus, we present the Natural Disasters Picture System (NDPS), a database of pictures of natural hazards and their impact on the environment and humans. Beyond the academic investigation, the objective of this work is to get a better understanding of natural hazard perception, with an emphasis on volcanic threats, in order to improve communication strategies during crises. In this article, we present the creation and implementation of the NDPS as well as the procedure and results of its validation among a population of Western European individuals. We also provide a distinctive emotional classification of the pictures and their contents.

Studies in the field of the affective sciences use different methods and materials to induce emotions, such as imagination, movie clips or static pictures. If emotions are to be genuinely elicited, the material has to be standardized and validated [1]. However, the affective sciences are not the only field that requires standardized material. Specific subject areas, such as the study of stereotypes based on perceptual cues (e.g., skin color, clothes) or risk behaviors in response to crises, also need such material in order to reduce biases inherent to stimulus characteristics. However, natural hazards are barely represented in the available databases. Let us consider one of the most widely used picture databases, the International Affective Picture System (IAPS) [2], which offers a large selection of pictures validated on the basis of appraisal dimensions. This database has been extensively used in the field of cognitive and affective science, such as in investigations of the psychological [3,4], psychopathological [5], and neurological [6] underpinnings of fear conditioning. The applications range from investigations of the fear system to depression, anxiety, psychophysiology, and emotion regulation, among many other fields of research, resulting in more than 3.700 citations. The variety of pictures ranges from positive (e.g., babies, landscapes, cute animals) to extremely negative pictures (e.g., mutilations, assaults, accidents), from low (e.g., lamps, spoons, bowls) to highly arousing pictures (e.g., aimed guns, mutilations, erotic couples), and from pictures inducing submissive (e.g., aimed guns, burn victims, attacks) to dominant perceptions (e.g., babies, flowers, cute animals). Despite this large selection of 956 pictures, natural hazards are barely represented in the IAPS (only 8 pictures: 5 of tornadoes, 1 of lightning, 1 of a volcano, 1 of a lava flow). This issue of the limited number of picture categories has already been pointed out by several studies (e.g., [7–9]). Consequently, three other validated image databases were recently published: the Emotional Picture System (377 pictures) [10], the Geneva Affective Picture Database (730 pictures) [7], and the Nencki Affective Picture System (1356 pictures) [9]. However, none of these databases included pictures of natural hazards. Considering the great diversity of natural hazards (e.g., earthquakes, tsunamis, tornadoes and on to volcanic eruptions), the affective and cognitive sciences require a comprehensive database, with a specific section devoted to volcanic hazards due to the variety of the related events (e.g., fumaroles, geysers, lahars, lava flows, pyroclastic flows, tephra falls).

In order to provide a database of pictures related to natural hazards, we first implemented the NDPS by selecting relevant pictures of several natural hazards (e.g., volcanic eruptions, earthquakes, hurricanes). To validate the NDPS, we then adopted a methodology somewhat similar to that used for the validation of the IAPS [2]. Specifically, we assessed the emotional aspects of the pictures using the Self-Assessment Manikin (SAM) scale [11], which is based on three dimensions taken from the appraisal theories [12,13]: arousal, valence and dominance. To these dimensions, we also added the assessment of certainty, given its significant weight in emotion discrimination, as has been mentioned by most authors working in the field of appraisal theories [14–17].

The certainty dimension reflects the probability of occurrence of an event and its outcome [12]. It is evaluated on the basis of how certain or uncertain an individual would feel about what is currently happening, and about what might follow. Certainty has shown itself to be a dimension with considerable discriminant power for the correct categorization of discrete emotions [12,13,15]. For example, fear and anger are negative emotions in terms of their valence and lead to a high level of arousal. However, they clearly differ as to their position on the certainty scale: while fear leads to a low perception of certainty, anger is related to a high perception of certainty. The “*Component Process Model”* [13,17] supports the idea that this component is of crucial importance in the pattern of appraisal evaluations, and highlights the need to make use of it if we wish to obtain a highly accurate categorization of discrete emotions. This is especially true for the basic emotions (i.e., anger, disgust, fear, sadness, happiness, and to a certain extent, surprise, interest, contempt) (for a review, see [18]), since each basic emotion gives rise to a specific pattern of appraisal [13]. As a consequence, adding certainty to the three dimensions from the SAM [11] should provide a more effective way of properly determining the specific discrete emotion induced by each picture of the NDPS. This also avoids the use of emotional labels as are found in the IAPS [8], which restricts the variety of the potentially elicited emotions. Indeed, such evaluations are limited to a selection of the basic emotions, thus giving rise to a large proportion of confounded or undifferentiated emotions (i.e., 263 of the 390 pictures rated). We believe studies in the affective and psychological sciences would benefit from a systematic evaluation of certainty, which is now considered to be a key element in judgments, decision-making and communication theories [19–23]. Researchers in fear-arousing communications [24,25], such as those related to natural disasters, require a validated fear-eliciting picture system.

In this article, we present the NDPS, a picture database exclusively dedicated to natural hazards and their impact on the environment and humans. Note that previous studies showed that cultural differences can lead to some various perceptions of natural hazards [26,27]. However, the current validation, as a first step of a broader validation, is based on a sample of participants from Western Europe with no prior experience and/or exposure to natural hazards. To evaluate each picture in the database, we used four dimensions: arousal, valence, dominance and certainty. We performed a two-stage validation operation involving a total of 120 participants, during which we divided the entire database into two sets of pictures. Finally, to provide a distinctive emotional classification of the pictures and their contents, we used a Hierarchical Ascendant Classification. Overall, this classification suggests four clusters depicting distinct patterns of emotions, subdivided into nine classes of picture types. The NDPS database is freely available together with the associated online material.

## Method

### Creation of the database

#### Description of the picture set

The database contains 154 pictures of various natural hazards (e.g., earthquakes, hurricanes, tornadoes), including an extended set of pictures of volcanic hazards (e.g., volcanic explosions, lava flows, ash and lapilli falls, and pyroclastic flows), aftermaths and victims. The readers should note that due to a copyright misattribution at the time of the searches, picture 19 had to be removed from our database upon validation. We used two sets of pictures, a first set of 72 pictures and a second set of 83 pictures. The characteristics of each picture are detailed in S1 Appendix. They include the picture identification number, its main category based on the nature of the hazard (e.g., earthquakes, lava flows), and three subcategories referred to as content (e.g., event, aftermath), kind of view (e.g., close-range, long-range), and specific features (e.g., aerial views).

Most pictures were taken from different web sources such as Flickr, Wikimedia Commons, USGS (United States Geological Survey), USDA (United States Department of Agriculture). The others were taken by various volcanologists (one is a co-author of the present article) or photographers. All pictures were either in the public domain or taken from Creative Commons, or permission was obtained from the author(s) for use as part of a database in a research context.

The database and on-line supporting material are freely available at: http://ndps.lapsco.fr.

#### Image processing

The photo quality varied depending on the source. We used Rawtherapee 4.0.11.8. to enhance the overall quality of the pictures. All of them were inspected and, if necessary, corrected in terms of focus quality, exposure (highlights, shadows and contrast), white-balance, resolution, and cropping. For example, to obtain a more natural effect, highlight/shadow corrections make it possible to compensate for over/underexposure. Most pictures were already high resolution (over 1200 x 800 pixels), except for pictures 50, 53, 54 and 65, which were only available in a lower resolution (800 x 500 pixels). The minimum resolution was finally set at around 1200 x 800 pixels for all pictures, thus giving rise to a homogenous and standardized database.

### Validation of the database

#### Participants and ethics statement

The participants consisted of 129 undergraduate students in Psychology from Clermont Auvergne University, formerly Blaise Pascal University (Clermont-Ferrand, France). We recruited 63 and 66 participants for the first and second sets of pictures, respectively. Participants who could not perform the task properly (n = 9) were excluded from the analysis for the following reasons: *(i)* did not pay attention to the change of order of the dimensions (n = 5), *(ii)* gave the same answer throughout the task (n = 1), *(iii)* could not understand how to use the dominance and certainty scales (n = 2), and *(iv)* suffered from Stargardt disease (n = 1) (Stargardt disease is a visual affliction defined as a macular degeneration which progressively reduces vision to the point of blindness, while leaving peripheral vision unaffected). This left us with a total of 120 participants (105 females, 15 males) with a mean age of 20.2±2.85 years. According to our methodology, 60 participants were assigned to each set, which resulted in the following sample specificities: The first set comprised 51 females and 9 males with a mean age of 20.1±3.07 while the second set comprised 54 females and 6 males with a mean age of 20.3±2.64.

The protocol was approved by the Ethics Committee of Clermont-Ferrand (ref: 2013/CE42). The nature of the study was fully explained and a written informed consent was obtained from each participant.

#### Dimensional evaluation

**Fig 1:**
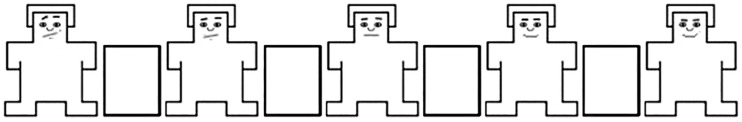
The additional dimension of certainty.

We used an enhanced version of the Self-Assessment Manikin (SAM) scale [11,28], the original form of which includes three basic dimensions (i.e., valence, arousal and dominance) from the appraisal theories [12,13]. For the purpose of this validation, we implemented a fourth dimension, namely certainty, by using similar illustrations designed with Gimp 2.8.6. (Fig 1). As for the SAM, each dimension involved a 9-point scale (i.e., five manikins, each spaced one step away from each other).

Given the results of the validation of the SAM [11], we were confident that the illustration would correspond to the explained evaluation, which can be summarized as follows: for the "uncertain" end of the scale, “*the picture makes you feel that you are uncertain of the situation and its outcome; that you do not know what is going on; that you are doubtful; that you cannot forecast the outcome; that you are unable to anticipate the future*”; for the "certain" end of the scale, “*the picture makes you feel that you are certain of the situation and its outcome; that you know what is going on; that you can forecast the outcome; that you are able to anticipate the future*”.

#### Procedure

The experimenter used standardized instructions, which were very similar to those used for the validation of the IAPS [2]. The purpose of the task was explained to participants and each of the four dimensions was then described. A form summarizing the instructions and a description of the dimensions were given to the participants in case they had any doubts during the task (S2 Appendix).

The task sequence was as follows. A preparation message “*Prepare yourself to evaluate the next picture*” appeared for 5 seconds on the screen, after which the picture appeared for 6 seconds. This was followed by the dimensional evaluation (enhanced SAM), during which the participants had 20 seconds to complete the four scales. If a participant could not finish in time, an alert message was displayed requesting him/her to complete the evaluation as quickly as possible. This specific time limit of 20 seconds was chosen to correspond to one used in the validation of the IAPS [2], in which participants had 15 seconds to complete three scales. The presentation order of the dimensions was varied during the task, and a special message informed the participant each time a new order occurred.

The task started with a training set of six randomly presented neutral pictures depicting natural hazards. The order of the dimensions changed between the third and fourth trial, and this was systematically pointed out by the experimenter. On the last two trials of the training set, the experimenter asked the participant to explain his or her choice for each dimension. In the event of any misunderstanding, the experimenter gave additional explanations before the start of the task. At the end of the training set, the experimenter answered any questions about the task, and the participant was left alone to complete the validation set.

For the validation set, the picture order was randomized. The order of presentation of the four dimensions changed four times during the task, as follows. First order: valence, arousal, dominance, certainty; second order: arousal, valence, certainty, dominance; third order: valence, certainty, dominance, arousal; fourth order: dominance, certainty, valence, and arousal. The change of order occurred every 18 pictures for the first set (picture 1 to 72), and every 21 pictures for the second set (picture 73 to 155). The task ran under E-Prime 2.0 (Psychology Software Tools, Pittsburg, PA) on a PC with a 17-inch screen (4:3). The entire protocol lasted around 45 minutes.

#### Statistical analysis

We calculated means and standard deviations for each dimension on each of the 154 pictures. We then performed Pearson correlations between each dimension for the overall sample. To examine the emotional patterns induced by the pictures, we conducted a Hierarchical Ascendant Classification (HAC) on the four dimensions, based on the distance between groups, using a solution ranging from one cluster up to the maximum number of clusters. To interpret the emotional pattern for each class, we calculated the means (±SD) of each dimension for each class. Statistical analyses were conducted using IBM SPSS 22 (IBM Corp., USA).

## Results

The means and standard deviations for each picture on each dimension are provided in S3 Appendix. Valence and arousal were negatively correlated, with the result that an appraisal of high arousal was associated with negative valence. A similar pattern was observed between arousal and dominance, as well as between arousal and certainty. Valence was positively correlated with dominance and certainty, with positive valence being associated with high dominance and certainty. Finally, dominance and certainty were positively correlated. All correlations are detailed in Table 1.

**Table 1:** Pearson’s correlation between dimensions for the whole sample.

	Valence	Arousal	Dominance	Certainty
Valence				
Arousal	-.85***(.72)			
Dominance	.75***(.56)	-.867***(.75)		
Certainty	.62***(.38)	-.758***(.58)	.75***(.56)	

*** p < .001; Numbers in brackets correspond to r^2^

The cluster classification revealed a coherent structure consisting of four main clusters (A, B, C, D), which can be decomposed into nine distinctive classes (I, II, …, IX). The classification is summarized in Table 2. For the purposes of consistency, we included the best-fitting emotions on the basis of the appraisal patterns and these are described in more detail in the section on discrete emotions (see below). S4 Appendix also presents the scatter plots for each correlation as a function of the clusters.

**Table 2:** Classification, mean dimension scores (±SD), and best-fitting emotion.

Cluster	Class	Valence	Arousal	Dominance	Certainty	Emotion
(A) Desolation	(I) Desolation/threat	4.62(0.18)	5.06(0.40)	4.33(0.26)	4.26(0.39)	Sadness
	(II) Desolation/loss	3.71(0.29)	5.35(0.40)	3.89(0.40)	4.43(0.41)	Sadness
(B) Neutral	(III) Neutral	5.46(0.32)	4.68(0.61)	4.60(0.54)	4.96(0.31)	Neutral/Interest
(C) Frightening	(IV) Shocking	2.56(0.36)	6.72(0.28)	2.42(0.22)	3.76(0.33)	Fear
	(V) Highly shocking	2.00(0.12)	7.36(0.26)	1.89(0.27)	3.60(0.60)	Fear
	(VI) Victims	1.70(0.23)	6.42(0.40)	3.06(0.31)	3.90(0.44)	Fear
	(VII) Aftermath/rubble	2.64(0.34)	5.91(0.44)	3.35(0.37)	4.46(0.22)	Fear
	(VIII) Volcanic eruption	3.47(0.23)	6.19(0.41)	2.81(0.34)	3.53(0.34)	Fear
(D) Pleasant/aesthetic	(IX) Pleasant/aesthetic	7.40(0.57)	2.94(0.40)	5.24(0.42)	6.15(0.39)	Aesthetic emotion

Standard deviations are in brackets. The class groups the different picture numbers.

Cluster (A) Desolation (2 classes, 46 pictures): Class I (14 pictures): Volcanic Eruptions (32, 33, 70), Acid lakes (44, 124), Active volcanoes (150, 155), Lava flows (100, 103), Geysers (46, 47), Aftermaths (107), Fumaroles (43), Lahars (49);

Class II (32 pictures): Aftermaths (5, 7, 9, 18, 27, 28, 40, 41, 51, 61, 65, 90, 94, 108, 111), Volcanic eruptions (30, 34, 36, 37, 60, 62, 69, 71), Lava flows (74, 101), Pyroclastic flows (66, 67), Avalanches (79), Volcanic ash clouds (63), Fumaroles (45), Lahars (50), Fires (83);

Cluster (B) Neutral (1 class, 7 pictures):

Class III: Avalanches (7 pictures): (1,2), Acid lakes (123), Lava flows (102), Sea storms (97), Volcanoes (152), Volcanic eruptions (42);

Cluster (C) Frightening (5 classes, 93 pictures):

Class IV (23 pictures): Lava flows (52, 53, 54, 104, 105, 110, 120, 121, 127), Fires (11, 12, 82, 87), Tornadoes (20, 21, 22), Floods (88, 89), Sea storm(98, 99), Lahars (112), Victim of pyroclastic flow (147), Volcanic explosions (38);

Class V (7 pictures): Fires (10, 39, 84, 86), Tornadoes (95, 96), Tsunamis (23);

Class VI (9 pictures): Human victims (133, 134, 135, 136, 140, 141, 142, 144), Cattle corpses (109);

Class VII (33 pictures): Aftermaths (3, 4, 6, 8, 13, 14, 15, 16, 17, 24, 25, 26, 29, 48, 64, 80, 81, 91, 92, 93, 114), Victims removed from rubble (132, 137, 138, 139, 143, 145, 146), Buildings on fire (85, 113), Lava flows (55, 56, 59);

Class VIII (21 pictures): Volcanic eruptions (31, 35, 72, 73, 75, 76, 77, 115, 116, 117, 118, 130, 131), Pyroclastic flows (128, 129, 68), Lava flows (57, 58, 106), Avalanches (78), Fumaroles (119);

Cluster (D) Pleasant/aesthetic (1 class, 8 pictures):

Class IX (8 pictures): Volcanoes (148, 149, 151, 153, 154), Acid lakes (122, 125, 126);

Cluster A comprised 46 pictures depicting (potential) desolation and had two different focal points. The first of these (Class I) was related to 14 threatening pictures, such as active geysers or active volcanoes close to a city. The second with 32 pictures (Class II) corresponded to loss, such as ruined houses, a badly damaged *Spiderman* doll, or a desolate landscape after a volcanic eruption. Cluster B included 7 pictures and was characterized by neutral pictures of different hazards, such as pictures of a prototypical (archetypal) volcano or sea waves. Cluster D encompassed 8 pictures and clearly differed from the others as it was related to aesthetically positive pictures of landscapes, such as volcanoes/mountains or colored acid lakes.

Cluster C consisted of 93 frightening pictures subdivided into five different classes (IV to VIII). Class IV (23 pictures) included shocking pictures of various hazards, while class V (7 pictures) comprised even more shocking pictures, such as a tsunami rushing toward people, a wildfire with fleeing animals, or a tornado destroying people's homes. Class VI (9 pictures) displayed victims (severe injuries, human corpses, or cattle corpses), and were therefore also highly shocking in a different way. The 33 pictures in Class VII mainly depicted post-disaster situations, with 85% of the pictures representing the aftermath of events and rubble or victims being removed from rubble. Of the remaining 15% of the pictures, two depicted buildings on fire, and three depicted lava flows. Class VIII (21 pictures) corresponded to scenes of volcanic eruptions and included pictures of lava. More specifically, 76% of the pictures showed volcanic eruptions, 14% lava flows, and a single picture represented a fumarole exhalation.

### Determination of the discrete emotions

Natural disasters are not specifically related to basic or discrete emotions, unlike most of the pictures from the IAPS (e.g., rotting food mainly associated with disgust, snakes mainly associated with fear). In order to determine the most probable emotion conveyed to participants by each cluster of pictures, we performed a cluster-based determination of the appraisal dimensions (valence, arousal, dominance, certainty) on the basis of the patterns of emotions known from appraisal theory [12,13,15] (Table 2).

We obtained two negative emotion patterns. The cluster *Desolation* (A) resulted in a negative valence (*M* = 3.99, *SD* = 0.49) coupled with low arousal (*M* = 5.26, *SD* = 0.42), low dominance (*M* = 4.02, *SD* = 0.41), and moderately low certainty (*M* = 4.38, *SD* = 0.41). This pattern was related to a negative emotion very close to sadness. The cluster *Frightening pictures* (C) resulted in a strong negative valence (*M* = 2.66, *SD* = 0.60), high arousal (*M* = 6.33, *SD* = 0.57), very low dominance (*M* = 2.86, *SD* = 0.56), and low certainty (*M* = 3.96, *SD* = 0.51). This pattern was mainly representative of fear.

In the case of positive emotions, happiness was expected to be associated with a highly positive valence, high arousal, dominance, and certainty. However, none of the pictures corresponded to such a pattern. The positive emotion observed in connection with the cluster *Pleasant/aesthetic* (D) was characterized by a highly positive valence (*M* = 7.40, *SD* = 0.57), very low arousal (*M* = 2.94, *SD* = 0.40), moderate dominance (*M* = 5.24, *SD* = 0.42), and high certainty (*M* = 6.15, *SD* = 0.39), and therefore corresponded to a more abstract category consisting of a pleasant aesthetic emotion or elation [13,29]. The cluster *Neutral* (B) was associated with moderate valence (*M* = 5.46, *SD* = 0.32), arousal (*M* = 4.68, *SD* = 0.61), dominance (*M* = 4.60, *SD* = 0.54) and certainty (*M* = 4.96, *SD* = 0.31), and therefore corresponded to an attitude of neutrality or to a minimum level of interest.

Overall, 89.6% of the pictures in the database were associated with negative emotions: 29.9% sadness (cluster *Desolation* A) and 60.4% fear (cluster *Frightening* C). Only 10.4% corresponded to positive emotions and neutral/interest, with 5.1% corresponding to an aesthetic emotion (cluster *Pleasant/aesthetic* D), and 4.9% to neutrality/interest (cluster *Neutral* B).

## Discussion

Here, we have presented the Natural Disasters Picture System (NDPS), a picture database dedicated to natural hazards and their impacts on the environment and humans. Regarding the implementation of the NDPS, we captured a wide variety of natural events such as snow avalanches, earthquakes, fires, floods, hurricanes, landslides, sea storms, tornadoes, tsunamis, and typhoons. Due to the great diversity of events arising from volcanic hazards, we also created a specific section relating to volcanic activity, such as acid lakes, fumaroles, geysers, lahars, lava flows, pyroclastic flows, tephra falls, and other volcanic phenomena. The results from the NDPS validation demonstrated that most of the pictures were of negative valence. Finally, the emotional classification analysis revealed that most of them induced emotions of fear or sadness. We have reported all the validation data for each picture and identified clusters.

The emotional aspects of this database mainly involved negative emotions, a finding consistent with our expectations concerning stimuli related to natural hazards. 90% of the pictures gave rise to negative emotions, which corresponded to fear and sadness. The classification revealed two very negative classes of pictures: the *Highly shocking* (V) and the *Victims* classes (VI). The remaining 10% of the pictures were found to elicit neutral and positive aesthetic emotions. In our opinion, these stimuli could be very useful for researchers, as protocols often require control conditions involving pictures of natural hazards that elicit positive emotions or convey neutrality rather than feelings of negative arousal. In addition to the positive aesthetic emotions, further studies should specifically examine how natural hazards could induce self-transcendent positive emotions, such as transcendence, calm, awe, and wonder (e.g., [30]), as such events could promote spiritual beliefs [31,32] and impact the culture of individuals living at risk of natural hazards such as volcanoes, earthquakes, and tsunamis.

We obtained a negative correlation between arousal and valence, with the most negative pictures leading to the highest arousal. This pattern was different to that obtained during the validation of the IAPS [2], in which the negative and positive pictures led to contrasting correlations between arousal and valence: negative pictures led to a negative correlation, whereas positive pictures led to a positive one. This specific effect was explained by the presence of a wide variety of emotions; ranging from positive/arousing pictures (e.g., happiness, sexual arousal) to positive/non-arousing pictures (e.g., interest), or from negative/arousing pictures (e.g., fear, anger) to negative/non-arousing pictures (e.g., sadness, boredom). However, all NDPS pictures were related to negative (i.e., fear, sadness) or positive/non-arousing (i.e., aesthetic emotion) emotions, but not to positive/arousing emotions. This would explain why we only found a negative relation between arousal and valence.

The positive correlations between dominance and certainty are consistent with the two negative emotions of fear and sadness [12,13,15]. Fear is characterized by low dominance and low certainty, whereas sadness is associated with low dominance and moderately low certainty [15]. We noticed that 60% of the pictures induced a perception of fear, thus leading to a strong positive correlation between dominance and certainty. The remaining 10% of the pictures, which induced a positive aesthetic emotion or neutrality, also involved a similar pattern of dominance and certainty. Sadness, which was found in response to 30% of the pictures, was the only emotion that did not involve a specific positive correlation between dominance and certainty. We therefore obtained a positive correlation between dominance and certainty for the whole sample of pictures.

The assessment of the certainty dimension allowed us to reduce the ambiguity in the identification of distinct emotional patterns. As we mentioned earlier, each emotion possesses a specific pattern of appraisal dimensions [12,15]. As far as the determination of emotions is concerned, the combination of valence and arousal is not in itself sufficient to permit discrimination. For instance, fear and anger share a similar pattern on these dimensions, as do sadness and boredom. However, in order to unambiguously discriminate between fear and anger, it is also necessary to evaluate certainty. Taken together, our results indicate the value of integrating the certainty dimension with the SAM in order to obtain suitable emotion discrimination.

Finally, the cluster classification resulted in coherent results that clearly mirrored the content of the pictures and the elicited emotions. The corresponding emotions that best matched the emotional patterns were consistent with the content of the pictures. For example, the pictures of desolation (e.g., devastated environment) led to patterns of sadness, while the pictures depicting an immediate threat to human beings (e.g., tsunami rushing toward people) aroused patterns of fear. The cluster C (*Frightening pictures)* contained some very potent classes, including *Victims* (VI), which portrayed severe injuries and corpses. A few of the pictures presented extremely shocking events (e.g., tsunami rushing toward people, forest fire with fleeing animals), and all of these were grouped in another *Highly shocking* (V) class. The other classes were also very coherent. For examples, the *Aftermath/rubble* (VIII) class depicted the aftermath of events, rubble or victims being removed from rubble, while the *Pleasant/aesthetic* (IV) class presented acid lakes with blue-tinted lagoons or picture-postcard (prototypical) volcanoes. The classification revealed by the NDPS therefore exhibited a high level of consistency within clusters and classes.

For the sake of clarity, we consider here some potential uses of the NDPS in experimental and applied psychology (evacuation of exposed population), cognitive neuroscience (investigation of the fear system during uncertain or natural disasters, etc.), and even applied volcanology. Complementarily, the IAPS [2] had a tremendous and extensive impact in psychology, cognitive neurosciences, and—more broadly—affective sciences, as evidenced by the number of citations (more than 3500 for the 2008 reference). However, a weakness of IAPS is that this database is constituted of general stimuli that cannot be applied to natural disasters. The NDPS can be used separately or in combination with the IAPS in studies requiring natural hazard stimuli (e.g., induction, context, natural hazard stimuli). Indeed, the NDPS used a similar validation protocol for the purpose of potential combined uses with the IAPS. The NDPS would also benefit applied psychology, such as in the field of communication, for which uncertainty remains important (e.g., uncertainty reduction, uncertainty management theories; for some reviews, see [23,33,34]), and even applied volcanology, about which uncertainty is a constant preoccupation [35]. For example, in information campaigns, scientists would be able to select congruent images with the level of uncertainty associated with the messages. These examples of the uses of the NDPS demonstrate some of its potential in these different fields of research. These NDPS application examples yield support for its potential in different research fields.

Regarding the main limitations of this study, we have to underline that our sample was composed of Western European students in psychology who had no prior experience and/or exposure to natural hazards. This constitutes both an advantage and a limitation: Unexposed individuals should provide an impartial—thus objective—view of the events, but they can also misperceive threats’ consequences. To improve the impact and utility of the NDPS, additional validations on populations with other cultural characteristics (e.g., eastern culture, population at risk of a specific natural hazard) are warranted as they may lead to some different perceptions.

## Conclusion

The NDPS provides a new tool for studying natural events and disasters in the field of affective and cognitive sciences, including a wide variety of natural events with a specific section related to volcanic activity. Its dimensional validation with four dimensions (SAM + certainty) means that this new database could be used for a range of research works, especially because the certainty dimension is of key importance in social cognition. The hierarchical classification also provides researchers with a coherent and distinctive emotional classification of the pictures and their features. In addition, the use of the NDPS is not restricted to scientific research applications as it could also find concrete applications in a range of communication purpose, such as in prevention or information campaigns. In its current stage, however, the NDPS relies on a sample of Western European individuals with no exposure to natural hazards. Further validation studies are still required to provide samples of individuals from different cultures and with exposure to natural hazards.

This database is freely available for use in scientific research and more information can be found at http://ndps.lapsco.fr.

## Supporting information

S1 Appendix Appendix I—Picture features. (PDF)

S2 Appendix Appendix II—Summarized form of the instructions given to participants. (PDF)

S3 Appendix Appendix III—Means and standard deviations for each picture on each dimension. (PDF)

S4 Appendix Appendix IV—Scatter plots for each correlation as a function of the clusters. (PDF)
