# Supplementary material for: Introduction and validation of the Natural Disasters Picture System (NDPS)
Source: PLoS One. 2018 Aug 8;13(8):e0201942. doi: 10.1371/journal.pone.0201942 (PMC6082542; doi:10.1371/journal.pone.0201942)
Supplement: S1 Appendix — (PDF) [file pone.0201942.s001.pdf]

Appendix I

Picture features

Natural Hazards				
No.	Main category	Subcategories		
		Content	Kind of view	Specific features
1	Avalanche	Event	Long-range	
2	Avalanche	Event	Long-range	
78	Avalanche	Event	Close-range	
79	Avalanche	Event	Close-range	
3	Earthquake	Event/Aftermath	Long-range	
4	Earthquake	Event/Aftermath	Long-range	
6	Earthquake	Event/Aftermath	Long-range	
5	Earthquake	Event/Aftermath	Close-range	
80	Earthquake	Event/Aftermath	Close-range	
81	Earthquake	Event/Aftermath	Close-range	
10	Fire	Event	Close-range	
11	Fire	Event	Long-range	
12	Fire	Event	Long-range	
82	Fire	Event	Close-range	
83	Fire	Event	Close-range	
84	Fire	Event	Long-range	
85	Fire	Event	Close-range	Firefighters
86	Fire	Event	Long-range	
87	Fire	Event	Close-range	Firefighting vehicle
7	Floods	Event	Long-range	
8	Floods	Event	Long-range	
9	Floods	Event	Close-range	
88	Floods	Event	Close-range	People
89	Floods	Event	Close-range	
97	Sea Storm	Event	Long-range	
98	Sea Storm	Event	Close-range	
99	Sea Storm	Event	Close-range	People
13	Hurricane	Aftermath	Long-range	
14	Hurricane	Aftermath	Long-range	
15	Hurricane	Aftermath	Long-range	
16	Hurricane	Aftermath	Long-range	
90	Hurricane	Aftermath	Close-range	High-impact loss
20	Tornado	Event	Long-range	
21	Tornado	Event	Long-range	
22	Tornado	Event	Long-range	
95	Tornado	Event	Long-range	Buildings
96	Tornado	Event	Long-range	Buildings
91	Tornado	Aftermath	Close-range	High-impact loss
92	Tornado	Aftermath	Close-range	High-impact loss
23	Tsunami	Event	Close-range	
24	Tsunami	Aftermath	Close-range	
25	Tsunami	Aftermath	Long-range	
26	Tsunami	Aftermath	Long-range	
27	Typhoon	Aftermath	Close-range	
28	Typhoon	Aftermath	Close-range	
29	Typhoon	Aftermath	Close-range	
17	Landslide	Event/Aftermath	Long-range	
18	Landslide	Event/Aftermath	Long-range	

Natural Hazards				
No.	Main category	Subcategories		
		Content	Kind of view	Specific features
93	Landslide	Event/Aftermath	Close-range	
94	Landslide	Event/Aftermath	Long-range	

Volcanic Hazards				
No.	Main category	Subcategories		
		Content	Kind of view	Specific features
52	Lava Flow	Event	Close-range	
53	Lava Flow	Event	Long-range	
54	Lava Flow	Event	Long-range	
55	Lava Flow	Event	Long-range	
56	Lava Flow	Event	Close-range	
57	Lava Flow	Event	Long-range	Lava lake
58	Lava Flow	Event	Close-range	Lava lake
59	Lava Flow	Event	Close-range	
74	Lava Flow	Event	Close-range	Boulder slide
104	Lava Flow	Event	Close-range	
105	Lava Flow	Event	Close-range	
106	Lava Flow	Event	Close-range	
120	Lava Flow	Event	Long-range	City
121	Lava Flow	Event	Long-range	Eruption
100	Lava Flow	Event	Close-range	People
101	Lava Flow	Event	Close-range	People
102	Lava Flow	Event	Close-range	People
103	Lava Flow	Event	Close-range	People
127	Lava Flow	Event	Close-range	Lava fall
39	Lava Flow	Aftermath	Close-range	
113	Lava Flow	Aftermath	Close-range	
110	Lava Flow	Aftermath	Close-range	
66	Pyroclastic Flow	Event	Long-range	
67	Pyroclastic Flow	Event	Long-range	
68	Pyroclastic Flow	Event	Long-range	
128	Pyroclastic Flow	Event	Close-range	
129	Pyroclastic Flow	Event	Close-range	
65	Pyroclastic Flow	Aftermath	Long-range	
40	Pyroclastic Flow	Aftermath	Close-range	
114	Pyroclastic Flow	Aftermath	Long-range	
43	Fumaroles	Event	Long-range	
45	Fumaroles	Event	Close-range	
119	Fumaroles	Event	Long-range	Large degassing
46	Geyser	Event	Close-range	
47	Geyser	Event	Long-range	
122	Acid Lake	Event	Close-range	
123	Acid Lake	Event	Close-range	
124	Acid Lake	Event	Close-range	
125	Acid Lake	Event	Long-range	
126	Acid Lake	Event	Long-range	
44	Acid Lake	Event	Close-range	Dallol
69	Eruption	Event	Long-range	Volcanic Plume
115	Eruption	Event	Long-range	Volcanic Plume
70	Eruption	Event	Long-range	Volcanic Plume

Volcanic Hazards				
No.	Main category	Subcategories		
		Content	Kind of view	Specific features
71	Eruption	Event	Long-range	Volcanic Plume
72	Eruption	Event	Close-range	Volcanic Plume
118	Eruption	Event	Long-range	Plume and lightning
42	Eruption	Event	Long-range	Phreatic plume
130	Eruption	Event	Long-range	Lava flow
131	Eruption	Event	Long-range	Strombolian
116	Eruption	Event	Close-range	Plume w. people
117	Eruption	Event	Close-range	Plume w. people
35	Eruption	Event	Close-range	Ballistic projectiles
36	Eruption	Event	Long-range	Ballistic projectiles
37	Eruption	Event	Long-range	Ballistic projectiles
73	Eruption	Event	Close-range	Ballistic projectiles
38	Eruption	Event	Long-range	Of high intensity
75	Eruption	Event	Long-range	Of high intensity
76	Eruption	Event	Long-range	Of high intensity
77	Eruption	Event	Long-range	Of high intensity
30	Eruption	Event	Long-range	Aerial Photo
31	Eruption	Event	Long-range	Aerial Photo
32	Eruption	Event	Long-range	Aerial Photo
33	Eruption	Event	Long-range	Aerial Photo
34	Eruption	Event	Long-range	Aerial Photo
60	Tephra falls	Event	Long-range	Volcanic plume
62	Tephra falls	Event	Long-range	Volcanic plume
63	Tephra falls	Event	Close-range	Fall-out
61	Tephra falls	Aftermath	Close-range	
64	Tephra falls	Aftermath	Long-range	
41	Tephra falls	Aftermath	Close-range	
107	Tephra falls	Aftermath	Close-range	
108	Tephra falls	Aftermath	Close-range	
109	Tephra falls	Aftermath	Close-range	
111	Tephra falls	Aftermath	Close-range	
49	Lahar	Event	Close-range	
50	Lahar	Event	Close-range	
51	Lahar	Aftermath	Long-range	
112	Lahar	Aftermath	Close-range	
48	Lahar	Aftermath	Close-range	
148	Volcano	Unprototypical		
149	Volcano	Unprototypical		
150	Volcano	Unprototypical		
151	Volcano	Unprototypical		
152	Volcano	Prototypical		
153	Volcano	Prototypical		
154	Volcano	Prototypical		
155	Volcano	Prototypical		

Victims				
No.	Main category	Subcategories		
		Content	Kind of view	Specific features
132	Victims	Survivor removed from rubble	Foreground	
133	Victims	Corpse	Foreground	
134	Victims	Corpse	Foreground	
135	Victims	Injured individual	Portrait	
136	Victims	Injured individual	Portrait	
137	Victims	Survivor removed from rubble	Foreground	
138	Victims	Survivor in rubble	Foreground	
139	Victims	Survivor removed from rubble	Foreground	
140	Victims	Hospital	Overview	
141	Victims	Survivor in rubble	Foreground	
142	Victims	Corpse	Foreground	
143	Victims	Survivor removed from rubble	Foreground	
144	Victims	Injured individual	Foreground	
145	Victims	Injured individual	Foreground	
146	Victims	Injured individual	Foreground	
147	Victims	Corpses	Overview	
