# Supplementary material for: Introduction and validation of the Natural Disasters Picture System (NDPS)
Source: PLoS One. 2018 Aug 8;13(8):e0201942. doi: 10.1371/journal.pone.0201942 (PMC6082542; doi:10.1371/journal.pone.0201942)
Supplement: S2 Appendix — (PDF) [file pone.0201942.s002.pdf]

Appendix II

Summarized form of the instructions given to participants

Valence: *Scales making it possible to describe intermediate levels*

Positive — Valence — Negative

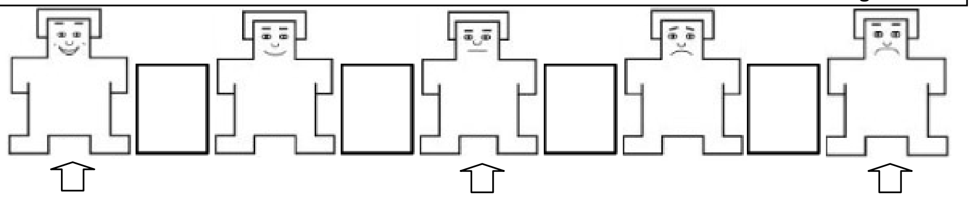

Here, the picture makes you feel cheerful, happy, joyful, content, pleased, hopeful, …

In the middle, you do not feel any of this; you are in a neutral state.

Here, the picture makes you feel sad, miserable, upset, annoyed, hopeless, …

Arousal: *Scales making it possible to describe intermediate levels*

Excited — Arousal — Calm

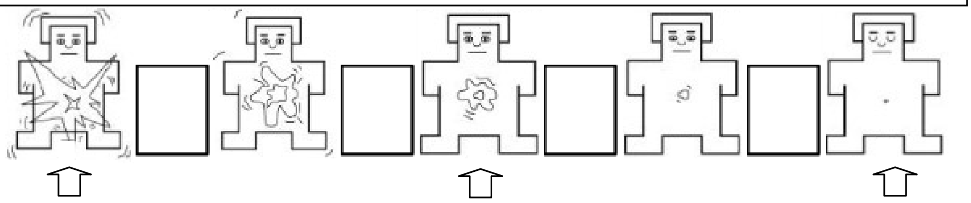

Here, the picture makes you feel excited, in a frenzy, nervous, sharp, awake, ...

In the middle, you do not feel any of this; you are in a neutral state.

Here, the picture makes you feel relaxed, calm, dull, sleepy, …

Dominance: *Scales making it possible to describe intermediate levels*

Dominated — Dominance — Dominant

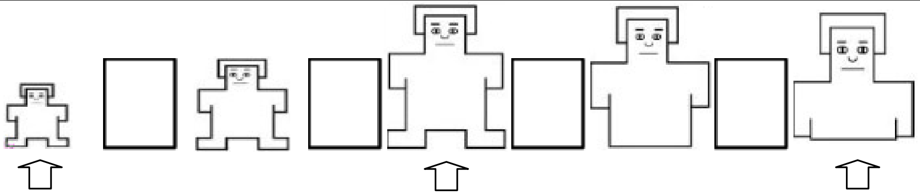

Here, the picture makes you feel that you are dominated; that you do not have any control; that you are under the influence; intimidated, submissive; that you feel small…

In the middle, you do not feel any of this; you are in a neutral state.

Here, the picture makes you feel that you are dominant; that you are in control; that you can influence the event; you are important, independent…

Certainty: *Scales making it possible to describe intermediate levels*

Uncertain — Certainty — Certain

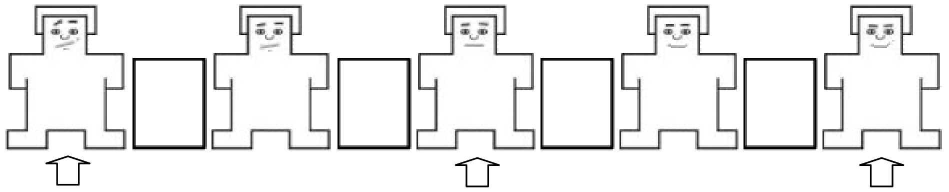

Here, the picture makes you feel that you are uncertain of the situation and its outcome; that you do not know what is going on; that you are doubtful; that you cannot forecast the outcome; that you are unable to anticipate the future.

In the middle, you do not feel any of this; you are in a neutral state.

Here, the picture makes you feel that you are certain of the situation and its outcome; that you know what is going on; that you can forecast the outcome; that you are able to anticipate the future.
