# Supplementary material for: Introduction and validation of the Natural Disasters Picture System (NDPS)
Source: PLoS One. 2018 Aug 8;13(8):e0201942. doi: 10.1371/journal.pone.0201942 (PMC6082542; doi:10.1371/journal.pone.0201942)
Supplement: S3 Appendix — (PDF) [file pone.0201942.s003.pdf]

Appendix III Means and standard deviations for each picture on each dimension

No.	Main Category	Valence Mean(SD)	Arousal Mean(SD)	Dominance Mean(SD)	Certainty Mean(SD)	Emotion	Cluster	Class
1	Avalanche	5.52(1.91)	4.73(1.89)	5.27(1.94)	5.40(2.04)	Neutral/Interest	Neutral (B)	Neutral (III)
2	Avalanche	5.62(1.87)	4.35(1.77)	4.76(2.02)	5.12(1.95)	Neutral/Interest	Neutral (B)	Neutral (III)
3	Earthquake	2.18(1.38)	6.10(2.02)	3.10(1.90)	4.53(2.26)	Fear	Frightening (C)	Aftermath/rubble (VII)
4	Earthquake	2.87(1.37)	5.48(1.86)	3.63(1.73)	4.55(2.05)	Fear	Frightening (C)	Aftermath/rubble (VII)
5	Earthquake	3.00(1.30)	5.27(1.84)	4.20(1.98)	5.27(2.02)	Sadness	Desolation(A)	Desolation/loss (II)
6	Earthquake	2.18(1.31)	5.98(1.85)	3.19(1.95)	4.63(2.34)	Fear	Frightening (C)	Aftermath/rubble (VII)
7	Floods	4.03(1.86)	4.93(1.67)	4.44(1.99)	4.82(1.87)	Sadness	Desolation (A)	Desolation/loss (II)
8	Floods	3.23(1.68)	5.33(1.75)	3.34(2.11)	4.93(2.24)	Fear	Frightening (C)	Aftermath/rubble (VII)
9	Floods	3.42(1.48)	5.25(1.56)	3.83(1.76)	5.12(2.19)	Sadness	Desolation (A)	Desolation/loss (II)
10	Fire	1.82(1.17)	7.70(1.57)	1.92(1.49)	3.95(2.91)	Fear	Frightening (C)	Highly shocking (V)
11	Fire	2.02(1.50)	7.03(1.58)	2.36(1.64)	3.82(2.84)	Fear	Frightening (C)	Shocking (IV)
12	Fire	2.28(1.62)	7.02(1.68)	2.58(1.86)	4.15(2.89)	Fear	Frightening (C)	Shocking (IV)
13	Hurricane	2.73(1.53)	5.67(1.82)	3.02(1.61)	4.35(2.17)	Fear	Frightening (C)	Aftermath/rubble (VII)
14	Hurricane	2.92(1.48)	5.32(2.10)	3.83(1.91)	4.58(1.88)	Fear	Frightening (C)	Aftermath/rubble (VII)
15	Hurricane	2.88(1.56)	6.05(1.97)	2.90(1.75)	4.13(2.53)	Fear	Frightening (C)	Aftermath/rubble (VII)
16	Hurricane	3.03(1.71)	5.65(2.01)	2.97(2.00)	4.58(2.26)	Fear	Frightening (C)	Aftermath/rubble (VII)
17	Landslide	2.53(1.40)	6.27(1.89)	2.76(1.88)	4.67(2.63)	Fear	Frightening (C)	Aftermath/rubble (VII)
18	Landslide	3.75(1.96)	5.30(2.04)	3.81(2.06)	4.52(2.00)	Sadness	Desolation (A)	Desolation/loss (II)
20	Tornado	2.68(1.80)	6.98(2.03)	2.31(1.68)	4.23(2.88)	Fear	Frightening (C)	Shocking (IV)
21	Tornado	2.95(1.57)	6.53(1.98)	2.61(1.52)	3.68(2.62)	Fear	Frightening (C)	Shocking (IV)
22	Tornado	2.58(1.58)	7.10(1.69)	2.24(1.63)	3.65(2.69)	Fear	Frightening (C)	Shocking (IV)
23	Tsunami	2.18(1.61)	7.57(1.62)	2.19(1.61)	3.77(2.91)	Fear	Frightening (C)	Highly shocking (V)
24	Tsunami	2.38(1.55)	5.70(2.08)	3.29(1.88)	4.42(2.42)	Fear	Frightening (C)	Aftermath/rubble (VII)
25	Tsunami	2.72(1.45)	5.30(2.09)	3.22(1.73)	4.30(2.17)	Fear	Frightening (C)	Aftermath/rubble (VII)
26	Tsunami	2.47(1.43)	5.80(2.02)	3.29(2.15)	4.63(2.28)	Fear	Frightening (C)	Aftermath/rubble (VII)
27	Typhoon	3.58(1.51)	5.20(1.95)	4.15(1.83)	4.67(2.19)	Sadness	Desolation (A)	Desolation/loss (II)
28	Typhoon	3.32(1.11)	5.18(1.60)	3.98(1.98)	4.63(2.07)	Sadness	Desolation (A)	Desolation/loss (II)
29	Typhoon	2.82(1.43)	6.10(1.70)	3.42(1.76)	4.47(2.28)	Fear	Frightening (C)	Aftermath/rubble (VII)
30	Eruption	4.02(2.27)	5.62(2.24)	3.54(2.14)	4.15(2.12)	Sadness	Desolation (A)	Desolation/loss (II)
31	Eruption	3.33(1.84)	6.35(1.80)	3.17(2.00)	3.73(2.27)	Fear	Frightening (C)	Volcanic eruption (VIII)
32	Eruption	4.42(1.67)	5.48(1.57)	4.42(1.87)	4.57(2.03)	Sadness	Desolation (A)	Desolation/threat (I)
33	Eruption	4.70(1.80)	4.83(1.93)	4.27(2.08)	4.33(1.99)	Sadness	Desolation (A)	Desolation/threat (I)
34	Eruption	3.73(1.47)	5.42(1.76)	3.68(2.05)	4.47(2.17)	Sadness	Desolation (A)	Desolation/loss (II)
35	Eruption	3.28(1.26)	6.08(1.89)	3.22(1.58)	3.65(1.86)	Fear	Frightening (C)	Volcanic eruption (VIII)
37	Eruption	3.62(1.67)	5.85(1.55)	3.34(1.61)	4.20(2.04)	Sadness	Desolation (A)	Desolation/loss (II)
38	Eruption	2.73(2.09)	6.85(1.88)	2.49(1.98)	3.85(2.75)	Fear	Frightening (C)	Shocking (IV)
39	Lava Flow	2.02(1.47)	7.42(1.57)	2.25(1.76)	4.52(2.83)	Fear	Frightening (C)	Highly shocking (V)

No.	Main Category	Valence Mean(SD)	Arousal Mean(SD)	Dominance Mean(SD)	Certainty Mean(SD)	Emotion	Cluster	Class
40	Pyroclastic Flow	3.85(1.87)	5.33(1.85)	4.32(2.10)	5.00(2.06)	Sadness	Desolation (A)	Desolation/loss (II)
41	Tephra falls	3.47(1.50)	4.65(1.92)	5.12(2.01)	5.18(2.35)	Sadness	Desolation (A)	Desolation/loss (II)
42	Eruption	5.15(1.63)	4.67(1.80)	4.81(1.62)	4.77(1.99)	Neutral/Interest	Neutral (B)	Neutral (III)
43	Fumarole	4.43(1.29)	5.08(1.87)	4.39(1.90)	3.90(1.63)	Sadness	Desolation (A)	Desolation/threat (I)
44	Acid Lake	4.78(1.88)	5.17(2.14)	4.44(1.73)	3.35(2.04)	Sadness	Desolation (A)	Desolation/threat (I)
45	Fumarole	3.90(1.41)	5.48(1.80)	3.90(1.49)	3.72(1.79)	Sadness	Desolation (A)	Desolation/loss (II)
46	Geyser	4.83(1.68)	5.47(1.81)	4.53(1.80)	4.00(2.07)	Sadness	Desolation (A)	Desolation/threat (I)
47	Geyser	4.37(1.40)	5.43(1.68)	4.29(1.60)	3.75(1.69)	Sadness	Desolation (A)	Desolation/threat (I)
48	Lahar	2.98(1.58)	6.03(1.77)	2.98(1.92)	4.03(2.28)	Fear	Frightening (C)	Aftermath/rubble (VII)
49	Lahar	4.43(1.49)	4.78(1.80)	5.00(1.73)	4.28(1.94)	Sadness	Desolation (A)	Desolation/threat (I)
50	Lahar	3.97(1.28)	5.37(1.78)	3.90(1.64)	3.78(1.97)	Sadness	Desolation (A)	Desolation/loss (II)
51	Lahar	3.38(1.53)	5.67(1.76)	3.54(2.02)	3.87(2.26)	Sadness	Desolation (A)	Desolation/loss (II)
52	Lava Flow	2.50(1.66)	6.78(1.91)	2.44(1.74)	4.10(2.69)	Fear	Frightening (C)	Shocking (IV)
53	Lava Flow	2.67(1.48)	6.68(1.51)	2.68(1.82)	4.38(2.62)	Fear	Frightening (C)	Shocking (IV)
54	Lava Flow	2.68(1.55)	6.63(1.75)	2.76(1.99)	4.33(2.63)	Fear	Frightening (C)	Shocking (IV)
55	Lava Flow	3.07(1.53)	5.93(1.64)	3.27(1.86)	4.57(2.15)	Fear	Frightening (C)	Aftermath/rubble (VII)
56	Lava Flow	3.08(1.68)	6.23(1.65)	3.81(2.25)	4.33(2.40)	Fear	Frightening (C)	Aftermath/rubble (VII)
57	Lava Flow	3.55(2.30)	7.12(1.81)	2.61(1.83)	2.78(2.36)	Fear	Frightening (C)	Volcanic eruption (VIII)
58	Lava Flow	3.68(1.92)	6.23(1.84)	3.05(1.64)	4.02(1.87)	Fear	Frightening (C)	Volcanic eruption (VIII)
59	Lava Flow	3.17(1.86)	5.97(1.74)	3.07(1.74)	4.43(2.35)	Fear	Frightening (C)	Aftermath/rubble (VII)
60	Tephra falls	3.72(1.68)	5.77(1.79)	3.54(1.91)	4.13(2.31)	Sadness	Desolation (A)	Desolation/loss (II)
61	Tephra falls	3.77(1.53)	4.23(1.91)	4.64(1.90)	4.67(1.89)	Sadness	Desolation (A)	Desolation/loss (II)
62	Tephra falls	4.22(1.66)	5.05(1.68)	4.10(1.73)	4.68(1.87)	Sadness	Desolation (A)	Desolation/loss (II)
63	Tephra falls	3.42(1.57)	5.78(1.98)	4.02(2.03)	4.10(2.29)	Sadness	Desolation (A)	Desolation/loss (II)
64	Tephra falls	2.68(1.31)	5.38(2.00)	3.58(1.83)	4.60(2.09)	Fear	Frightening (C)	Aftermath/rubble (VII)
65	Pyroclastic Flow	3.90(1.50)	4.78(1.66)	4.07(1.62)	4.50(1.94)	Sadness	Desolation (A)	Desolation/loss (II)
66	Pyroclastic Flow	3.50(1.51)	5.72(1.53)	3.36(1.74)	4.42(2.22)	Sadness	Desolation (A)	Desolation/loss (II)
67	Pyroclastic Flow	3.87(1.55)	5.32(1.76)	3.81(1.70)	4.10(2.09)	Sadness	Desolation (A)	Desolation/loss (II)
68	Pyroclastic Flow	3.38(1.78)	6.23(1.92)	3.34(1.88)	4.10(2.12)	Fear	Frightening (C)	Volcanic eruption (VIII)
69	Eruption	4.02(1.88)	5.43(1.94)	3.61(1.83)	4.38(2.21)	Sadness	Desolation (A)	Desolation/loss (II)
70	Eruption	4.50(1.81)	4.72(1.89)	4.24(2.05)	4.68(2.10)	Sadness	Desolation (A)	Desolation/threat (I)
71	Eruption	3.80(1.95)	5.57(1.75)	3.51(1.83)	4.22(1.98)	Sadness	Desolation (A)	Desolation/loss (II)
72	Eruption	3.35(1.79)	6.22(1.82)	2.97(1.81)	4.00(2.36)	Fear	Frightening (C)	Volcanic eruption (VIII)
73	Eruption	3.98(1.88)	6.20(1.73)	2.75(1.61)	3.68(2.14)	Fear	Frightening (C)	Volcanic eruption (VIII)
74	Lava Flow	3.98(1.69)	6.07(1.94)	3.65(1.89)	3.98(2.23)	Sadness	Desolation (A)	Desolation/loss (II)
75	Eruption	3.42(1.65)	6.10(1.87)	2.87(1.60)	3.18(1.86)	Fear	Frightening (C)	Volcanic eruption (VIII)
76	Eruption	3.50(1.63)	5.97(1.77)	2.38(1.65)	3.47(2.18)	Fear	Frightening (C)	Volcanic eruption (VIII)
77	Eruption	3.17(1.40)	6.37(1.90)	2.43(1.28)	3.43(2.21)	Fear	Frightening (C)	Volcanic eruption (VIII)
78	Avalanche	3.72(1.98)	5.92(2.06)	2.43(1.90)	3.63(2.50)	Fear	Frightening (C)	Volcanic eruption (VIII)

No.	Main Category	Valence Mean(SD)	Arousal Mean(SD)	Dominance Mean(SD)	Certainty Mean(SD)	Emotion	Cluster	Class
79	Avalanche	4.32(2.00)	5.33(1.97)	3.48(2.25)	3.93(2.30)	Sadness	Desolation (A)	Desolation/loss (II)
80	Earthquake	2.60(1.26)	5.77(1.87)	3.33(1.64)	4.40(2.44)	Fear	Frightening (C)	Aftermath/rubble (VII)
81	Earthquake	2.43(1.17)	5.87(1.76)	3.23(1.88)	4.18(2.60)	Fear	Frightening (C)	Aftermath/rubble (VII)
82	Fire	2.27(1.27)	6.68(1.82)	2.33(1.73)	3.70(2.76)	Fear	Frightening (C)	Shocking (IV)
83	Fire	3.68(1.69)	5.33(1.87)	4.23(2.35)	4.45(2.23)	Sadness	Desolation (A)	Desolation/loss (II)
84	Fire	1.98(1.31)	7.55(1.58)	1.72(1.24)	3.78(3.13)	Fear	Frightening (C)	Highly shocking (V)
85	Fire	2.15(1.40)	6.93(1.66)	3.10(2.15)	4.35(2.75)	Fear	Frightening (C)	Aftermath/rubble (VII)
86	Fire	1.95(1.27)	7.12(1.55)	1.95(1.42)	3.05(2.46)	Fear	Frightening (C)	Highly shocking (V)
87	Fire	2.28(1.15)	6.58(1.63)	2.80(1.68)	3.53(2.38)	Fear	Frightening (C)	Shocking (IV)
88	Floods	2.28(1.12)	6.43(1.78)	2.30(1.34)	3.72(2.42)	Fear	Frightening (C)	Shocking (IV)
89	Floods	2.60(1.42)	6.62(1.84)	2.02(1.27)	3.28(2.27)	Fear	Frightening (C)	Shocking (IV)
90	Hurricane	3.35(1.40)	5.48(2.06)	3.95(2.01)	4.38(2.14)	Sadness	Desolation (A)	Desolation/loss (II)
91	Tornado	2.83(1.43)	5.07(2.07)	3.20(1.67)	4.38(2.31)	Fear	Frightening (C)	Aftermath/rubble (VII)
92	Tornado	2.47(1.13)	5.82(1.94)	3.20(1.84)	4.70(2.51)	Fear	Frightening (C)	Aftermath/rubble (VII)
93	Landslide	2.55(1.05)	5.98(1.83)	2.72(1.43)	4.30(2.09)	Fear	Frightening (C)	Aftermath/rubble (VII)
94	Landslide	3.65(1.95)	5.22(2.23)	3.73(2.20)	4.27(2.49)	Sadness	Desolation (A)	Desolation/loss (II)
95	Tornado	1.90(1.20)	7.13(1.76)	1.55(1.17)	3.45(2.90)	Fear	Frightening (C)	Highly shocking (V)
96	Tornado	2.12(1.28)	7.07(1.59)	1.63(0.99)	2.68(2.38)	Fear	Frightening (C)	Highly shocking (V)
97	Sea Storm	5.77(2.23)	5.72(2.22)	3.50(2.11)	4.82(2.05)	Neutral/Interest	Neutral (B)	Neutral (III)
98	Sea Storm	3.30(1.68)	6.82(1.62)	2.32(1.30)	3.90(2.64)	Fear	Frightening (C)	Shocking (IV)
99	Sea Storm	2.90(1.57)	6.72(1.81)	2.53(1.65)	4.05(2.38)	Fear	Frightening (C)	Shocking (IV)
100	Lava Flow	4.57(1.80)	5.65(2.00)	4.05(2.06)	4.60(2.16)	Sadness	Desolation (A)	Desolation/threat (I)
101	Lava Flow	3.77(1.65)	6.07(1.82)	3.45(2.08)	3.98(2.13)	Sadness	Desolation (A)	Desolation/loss (II)
102	Lava Flow	4.88(1.80)	5.18(1.94)	4.75(2.33)	5.27(2.43)	Neutral/Interest	Neutral (B)	Neutral (III)
103	Lava Flow	4.65(1.66)	5.55(1.86)	4.03(2.07)	4.45(2.17)	Sadness	Desolation (A)	Desolation/threat (I)
104	Lava Flow	2.85(1.53)	6.78(1.53)	2.45(1.47)	3.52(2.07)	Fear	Frightening (C)	Shocking (IV)
105	Lava Flow	2.38(1.44)	6.77(1.78)	2.23(1.52)	3.68(2.73)	Fear	Frightening (C)	Shocking (IV)
106	Lava Flow	3.32(1.63)	5.95(1.70)	2.98(1.78)	3.68(2.14)	Fear	Frightening (C)	Volcanic eruption (VIII)
107	Tephra falls	4.53(1.98)	4.75(1.72)	4.25(1.92)	4.72(2.29)	Sadness	Desolation (A)	Desolation/threat (I)
108	Tephra falls	3.35(1.34)	4.98(1.80)	4.27(2.05)	4.92(1.95)	Sadness	Desolation (A)	Desolation/loss (II)
109	Tephra falls	2.07(1.15)	5.88(1.88)	3.18(1.52)	4.20(2.32)	Fear	Frightening (C)	Victims (VI)
110	Lava Flow	1.95(1.02)	5.95(1.98)	2.42(1.57)	3.73(2.47)	Fear	Frightening (C)	Shocking (IV)
111	Tephra falls	3.82(1.59)	4.77(1.87)	3.67(1.72)	4.87(2.07)	Sadness	Desolation (A)	Desolation/loss (II)
112	Lahar	2.23(1.06)	6.25(1.88)	2.37(1.47)	3.77(2.23)	Fear	Frightening (C)	Shocking (IV)
113	Lava Flow	2.43(1.14)	6.47(1.55)	3.20(1.77)	4.68(2.43)	Fear	Frightening (C)	Aftermath/rubble (VII)
114	Pyroclastic Flow	3.23(1.47)	5.07(1.89)	3.28(1.58)	4.23(2.17)	Fear	Frightening (C)	Aftermath/rubble (VII)
115	Eruption	3.23(1.76)	6.10(1.84)	2.53(1.42)	3.23(1.81)	Fear	Frightening (C)	Volcanic eruption (VIII)
116	Eruption	3.50(1.43)	5.72(1.74)	3.25(1.54)	3.62(2.01)	Fear	Frightening (C)	Volcanic eruption (VIII)
117	Eruption	3.73(1.80)	5.87(1.89)	3.07(2.01)	3.55(2.27)	Fear	Frightening (C)	Volcanic eruption (VIII)

No.	Main Category	Valence Mean(SD)	Arousal Mean(SD)	Dominance Mean(SD)	Certainty Mean(SD)	Emotion	Cluster	Class
118	Eruption	3.12(1.98)	7.25(1.69)	2.15(1.57)	2.83(2.01)	Fear	Frightening (C)	Volcanic eruption (VIII)
119	Fumaroles	3.50(1.60)	5.90(1.79)	3.12(1.93)	3.33(2.16)	Fear	Frightening (C)	Volcanic eruption (VIII)
120	Lava Flow	2.85(1.71)	7.13(1.76)	2.53(1.70)	3.55(2.73)	Fear	Frightening (C)	Shocking (IV)
121	Lava Flow	2.88(1.54)	6.53(1.58)	2.65(1.68)	3.18(1.89)	Fear	Frightening (C)	Shocking (IV)
122	Acid Lake	8.18(1.23)	2.55(2.26)	6.15(2.26)	6.60(2.57)	Aesthetic emotion	Pleasant/aesthetic (D)	Pleasant/aesthetic (IX)
123	Acid Lake	5.68(2.29)	4.23(2.17)	4.63(2.25)	4.85(2.77)	Neutral/Interest	Neutral (B)	Neutral (III)
124	Acid Lake	4.83(2.42)	4.82(2.09)	4.53(2.26)	4.55(2.52)	Sadness	Desolation (A)	Desolation/threat (I)
125	Acid Lake	7.03(1.60)	3.53(2.31)	5.08(2.10)	5.63(2.11)	Aesthetic emotion	Pleasant/aesthetic (D)	Pleasant/aesthetic (IX)
126	Acid Lake	7.82(1.55)	2.70(2.23)	5.38(2.50)	6.47(2.17)	Aesthetic emotion	Pleasant/aesthetic (D)	Pleasant/aesthetic (IX)
127	Lava Flow	3.00(1.79)	7.07(1.57)	1.87(1.16)	3.52(2.57)	Fear	Frightening (C)	Shocking (IV)
128	Pyroclastic Flow	3.45(1.16)	5.52(1.92)	3.00(1.48)	3.40(1.89)	Fear	Frightening (C)	Volcanic eruption (VIII)
129	Pyroclastic Flow	3.23(1.42)	6.20(1.74)	2.55(1.47)	3.28(2.01)	Fear	Frightening (C)	Volcanic eruption (VIII)
130	Eruption	3.43(1.57)	6.17(1.62)	2.73(1.67)	3.77(2.20)	Fear	Frightening (C)	Volcanic eruption (VIII)
131	Eruption	3.92(2.11)	6.63(1.98)	2.40(1.75)	3.70(2.38)	Fear	Frightening (C)	Volcanic eruption (VIII)
132	Victims	2.40(1.83)	6.57(1.70)	3.53(2.13)	4.20(2.44)	Fear	Frightening (C)	Aftermath/rubble (VII)
133	Victims	1.73(0.97)	6.47(2.32)	3.13(2.17)	4.00(2.76)	Fear	Frightening (C)	Victims (VI)
134	Victims	1.33(0.63)	6.30(2.27)	2.28(1.76)	4.63(2.97)	Fear	Frightening (C)	Victims (VI)
135	Victims	1.37(0.66)	6.88(1.95)	3.20(2.33)	4.20(2.55)	Fear	Frightening (C)	Victims (VI)
136	Victims	1.65(0.94)	6.82(1.95)	3.32(2.09)	3.82(2.17)	Fear	Frightening (C)	Victims (VI)
137	Victims	2.52(1.77)	6.53(1.33)	3.62(1.86)	4.48(2.32)	Fear	Frightening (C)	Aftermath/rubble (VII)
138	Victims	2.18(1.17)	5.77(1.98)	3.58(2.17)	4.38(2.35)	Fear	Frightening (C)	Aftermath/rubble (VII)
139	Victims	2.27(1.35)	6.42(1.58)	3.47(2.30)	4.05(2.57)	Fear	Frightening (C)	Aftermath/rubble (VII)
140	Victims	1.85(1.39)	6.23(2.11)	3.15(2.01)	3.72(2.51)	Fear	Frightening (C)	Victims (VI)
141	Victims	1.85(1.12)	5.88(1.92)	3.17(1.98)	3.87(2.45)	Fear	Frightening (C)	Victims (VI)
142	Victims	1.63(1.02)	6.95(1.69)	2.93(2.15)	3.07(2.35)	Fear	Frightening (C)	Victims (VI)
143	Victims	2.60(1.51)	6.08(1.54)	3.95(2.20)	4.67(2.58)	Fear	Frightening (C)	Aftermath/rubble (VII)
144	Victims	1.78(1.28)	6.37(1.85)	3.18(2.17)	3.62(2.73)	Fear	Frightening (C)	Victims (VI)
145	Victims	2.22(1.21)	5.97(1.75)	4.20(2.31)	4.35(2.48)	Fear	Frightening (C)	Aftermath/rubble (VII)
146	Victims	2.15(1.25)	6.45(1.84)	4.32(2.35)	4.93(2.19)	Fear	Frightening (C)	Aftermath/rubble (VII)
147	Victims	1.93(1.23)	6.50(1.77)	2.45(1.73)	3.25(2.41)	Fear	Frightening (C)	Shocking (IV)
148	Volcano	6.55(2.13)	3.60(2.16)	4.78(2.41)	5.58(2.29)	Aesthetic emotion	Pleasant/aesthetic (D)	Pleasant/aesthetic (IX)
149	Volcano	7.17(1.89)	2.88(1.97)	4.87(2.34)	6.03(2.36)	Aesthetic emotion	Pleasant/aesthetic (D)	Pleasant/aesthetic (IX)
150	Volcano	4.67(1.74)	4.43(1.82)	4.20(2.02)	4.22(2.17)	Sadness	Desolation (A)	Desolation/threat (I)
151	Volcano	7.05(1.81)	2.67(1.77)	5.18(2.51)	6.23(2.17)	Aesthetic emotion	Pleasant/aesthetic (D)	Pleasant/aesthetic (IX)
152	Volcano	5.63(1.89)	3.90(2.00)	4.48(2.09)	4.52(2.32)	Neutral/Interest	Neutral (B)	Neutral (III)
153	Volcano	8.08(1.24)	2.77(2.14)	5.13(2.40)	6.53(2.21)	Aesthetic emotion	Pleasant/aesthetic (D)	Pleasant/aesthetic (IX)
154	Volcano	7.33(1.58)	2.83(2.12)	5.32(2.42)	6.10(2.26)	Aesthetic emotion	Pleasant/aesthetic (D)	Pleasant/aesthetic (IX)
155	Volcano	4.92(1.89)	4.62(2.11)	4.02(2.02)	4.30(2.07)	Sadness	Desolation (A)	Desolation/threat (I)
