# Supplementary material for: Introduction and validation of the Natural Disasters Picture System (NDPS)
Source: PLoS One. 2018 Aug 8;13(8):e0201942. doi: 10.1371/journal.pone.0201942 (PMC6082542; doi:10.1371/journal.pone.0201942)

Appendix IV

Scatter plots for each correlation as a function of the clusters

Correlation between *valence* and *arousal* as a function of classification

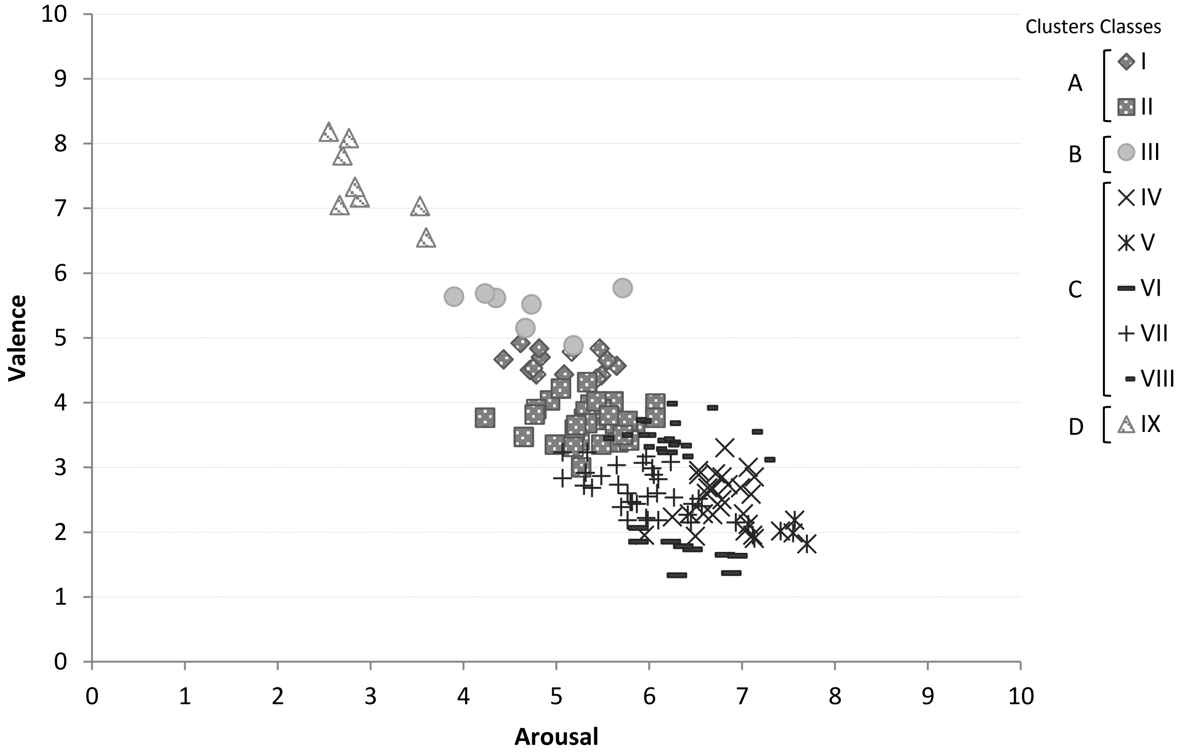

Correlation between *valence* and *dominance* as a function of classification

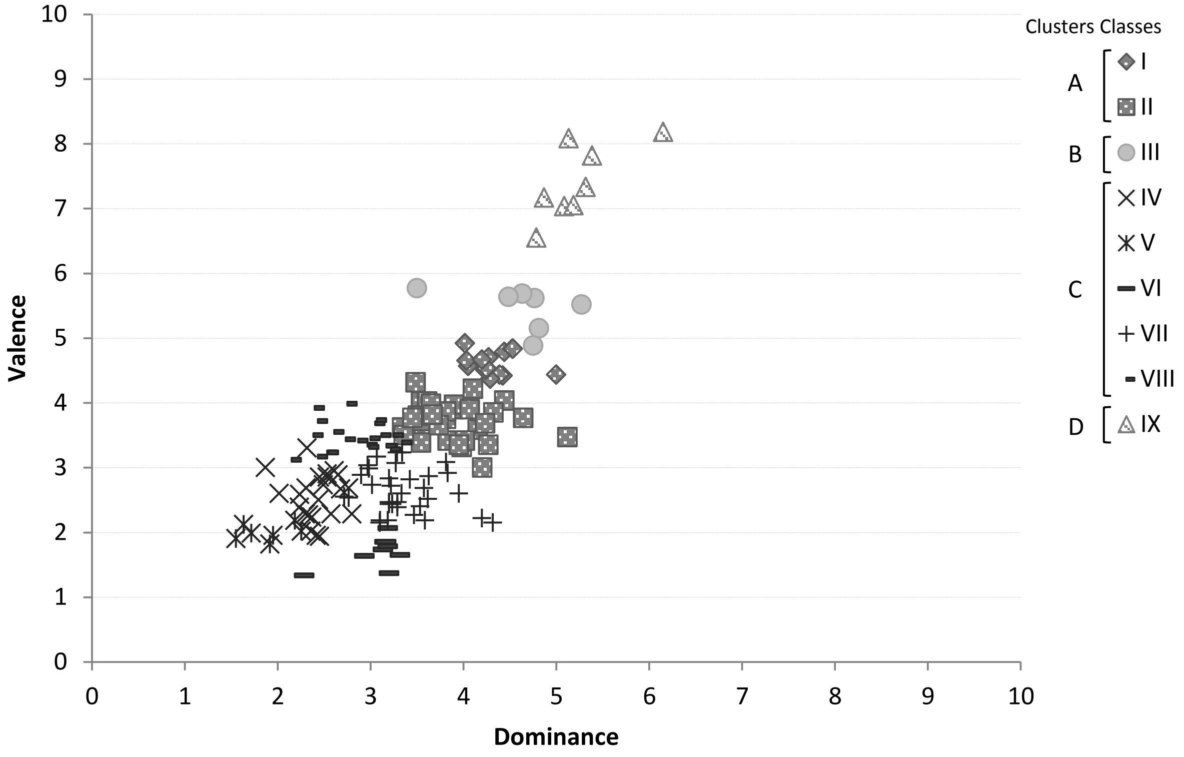

Correlation between *valence* and *certainty* as a function of classification

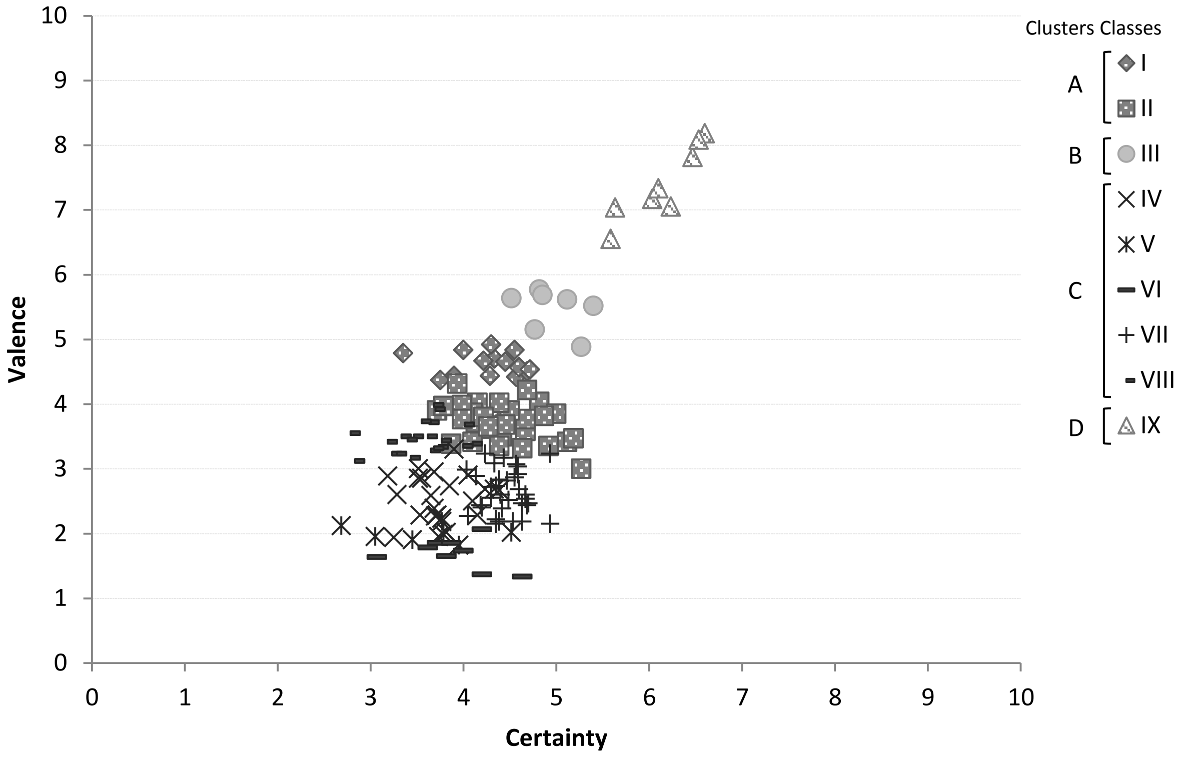

Correlation between *arousal* and *dominance* as a function of classification

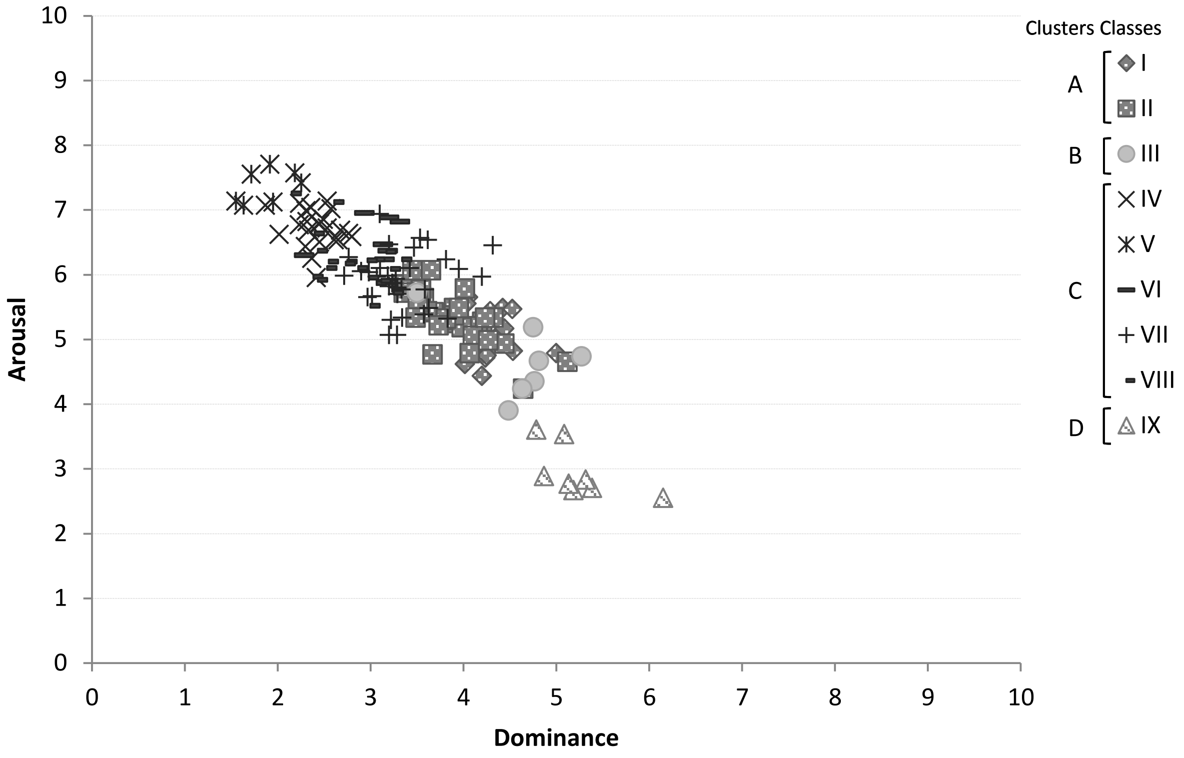

Correlation between *arousal* and *certainty* as a function of classification

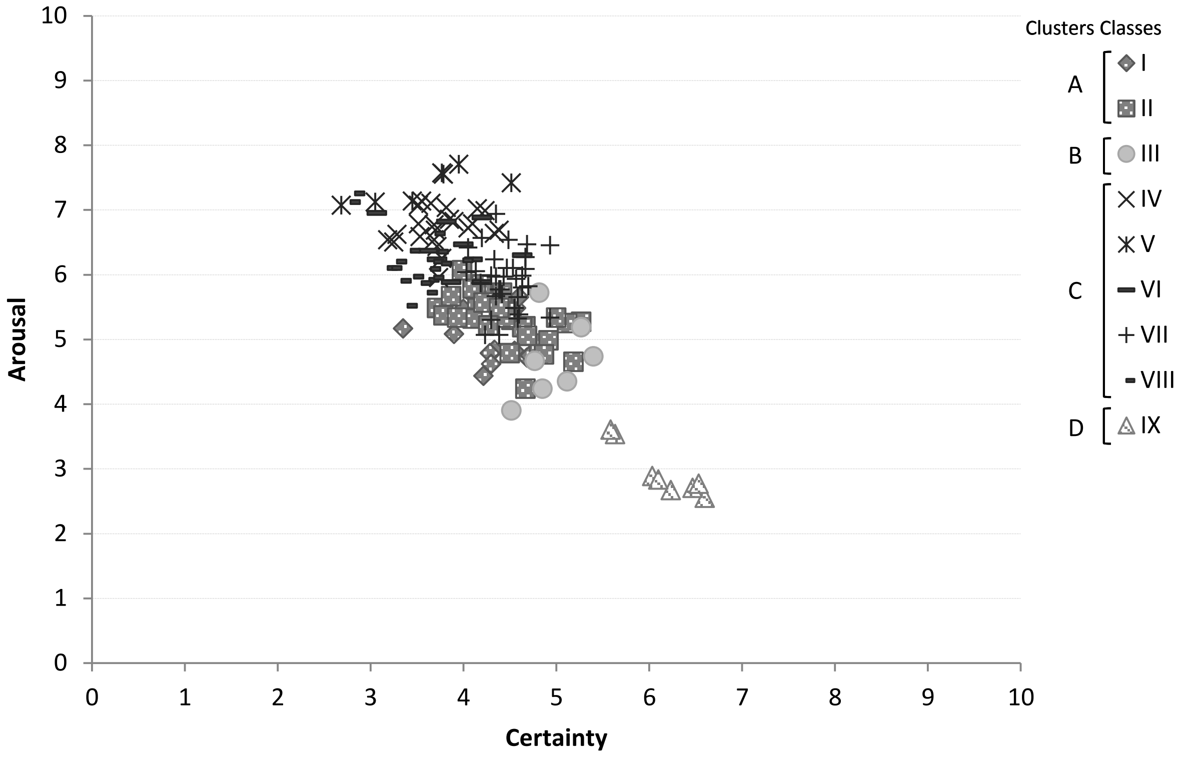

Correlation between *dominance* and *certainty* as a function of classification

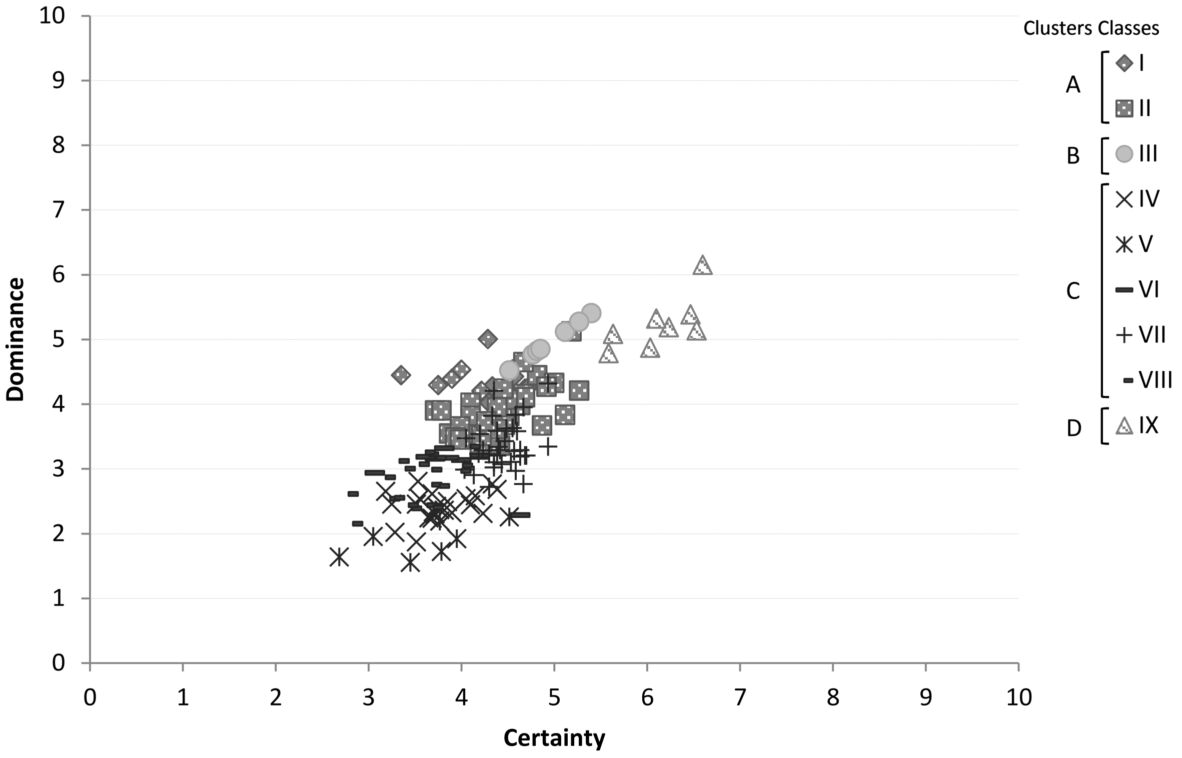

Supplement: S4 Appendix — (PDF) [file pone.0201942.s004.pdf]
